# Chelerythrine Chloride Alleviated Lipopolysaccharide-Induced Acute Lung Injury by Inhibiting Glycolytic Pathway Through Targeting Glyceraldehyde-3-Phosphate Dehydrogenase

**DOI:** 10.3390/molecules30122572

**Published:** 2025-06-12

**Authors:** Yuting He, Tianyun Fan, Ruishen Zhuge, Huiying Li, Guanjun Li, Lirun Zhou, Liting Xu, Xiaojiang Hao, Wei Gu, Jigang Wang

**Affiliations:** 1 State Key Laboratory of Discovery and Utilization of Functional Components in Traditional Chinese Medicine, Guizhou Medical University, Guiyang 550014, China; 2 Natural Products Research Center of Guizhou Province, Guiyang 550014, China; 3 State Key Laboratory for Quality Ensurance and Sustainable Use of Dao-di Herbs, Artemisinin Research Center, and Institute of Chinese Materia Medica, China Academy of Chinese Medical Sciences, Beijing 100700, China

**Keywords:** chelerythrine chloride, *Chelidonium majus* L., acute lung injury, glycolysis, glyceraldehyde-3-phosphate dehydrogenase

## Abstract

Acute lung injury (ALI) is a fatal respiratory disease caused by excessive inflammation. Chelerythrine chloride (CH), an isoquinoline alkaloid, exhibits diverse biological activities. The research focused on assessing CH’s therapeutic effects against LPS-mediated ALI in mice and its underlying mechanisms. The anti-inflammatory effects of CH were evaluated both in LPS-induced RAW264.7 cells and ALI mouse model. An amount of 2.5 μM CH significantly inhibited the secretion of nitric oxide (NO), tumor necrosis factor-α (TNF-α), interleukin-6 (IL-6) and IL-1β in RAW264.7 cells. CH treatment notably mitigated the thickened alveolar septa and reduced edema in LPS-induced ALI in mice. Activity-based protein profiling (ABPP) technology was employed to identify the targets of CH. Glyceraldehyde-3-phosphate dehydrogenase (GAPDH) was one of the direct targets of CH identified by ABPP. CH could downregulate the production of pyruvate. Furthermore, CH reduced the extracellular acidification rate (ECAR) while increasing the oxygen consumption rate (OCR) in LPS-stimulated RAW264.7 cells. All results suggest that CH mitigates LPS-induced ALI by targeting GAPDH and inhibiting glycolysis. This study reveals preliminary anti-inflammatory mechanisms of CH and its therapeutic potential for ALI.

## 1. Introduction

Inflammation is a fundamental protective mechanism of the immune system, activated in response to detrimental agents, such as viral and bacterial infections, toxicants, noxious chemicals and cellular injury [1,2]. While it typically functions as a protective mechanism, excessive or uncontrolled inflammation can result in severe organ damage [3,4]. Acute lung injury (ALI) represents a severe respiratory condition featuring abrupt onset, widespread alveolar injury, and refractory hypoxemia [5,6]. Globally, ALI affects approximately 3 million people annually, representing 10% of ICU admissions, and it carries a mortality rate of 30% to 50% [7]. Currently, the treatment for ALI primarily involves supportive measures, including nutritional support, infection control, ventilation strategies, and appropriate antimicrobial therapy, but these methods are not specific treatments for the inflammatory response in ALI [8]. Inflammatory responses are critically regulated by macrophage-derived mediators, including nitric oxide (NO), tumor necrosis factor (TNF)-α, interleukin (IL)-6, IL-1β and others, which collectively drive the progression of inflammatory cascades [9]. Although non-steroidal drugs and glucocorticoids can mitigate inflammation, their clinical use is frequently restricted by adverse effects, such as gastrointestinal or metabolic complications [10]. Therefore, the development of novel, effective anti-inflammatory therapies for ALI remains an urgent and unmet medical need.

Glycolysis serves as a fundamental metabolic pathway for the body’s energy supply and the production of intermediates [11]. Macrophage activation and immune function are closely regulated by glycolysis, and modulating this process can effectively reduce inflammation while enhancing immune responses [12]. During the early inflammatory phase, pro-inflammatory M1 macrophages are activated, exhibiting increased glycolytic activity to convert glucose into energy and promote the secretion of inflammatory cytokines [13]. Thus, targeting glycolysis in immune cells shows therapeutic potential in inflammatory conditions. As a key glycolytic enzyme, glyceraldehyde-3-phosphate dehydrogenase (GAPDH) mediates the conversion of glyceraldehyde-3-phosphate into 1,3-bisphosphoglycerate during glycolysis [14,15]. Elevated GAPDH activity is a hallmark of activated macrophages [16], rendering it a potential therapeutic target for inflammatory disorders.

**Figure 1:**
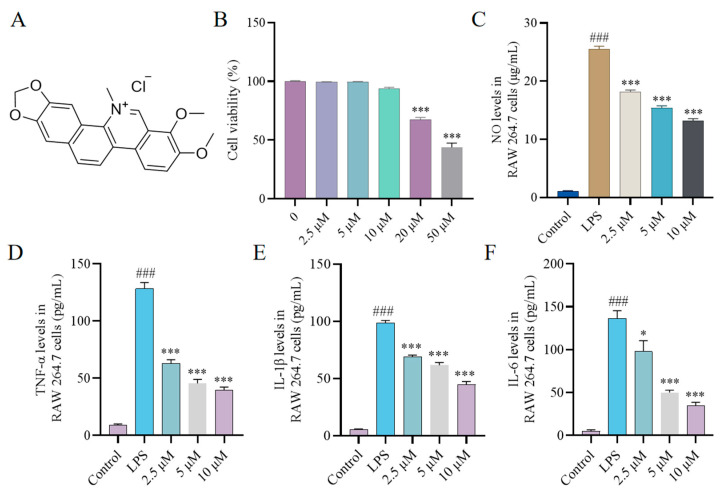
(**A**) The molecular structure of CH. (**B**) Cytotoxicity of CH on RAW264.7 cells. (**C**–**F**) Inhibitory effect of CH on NO, TNF-α, IL-1β and IL-6 release. ### *p* < 0.001 vs. Control group; * *p* < 0.05, *** *p* < 0.001 vs. LPS group. All experimental procedures were conducted with a minimum of three independent replicates.

*Chelidonium majus* L., a medicinal plant widely utilized in traditional medicine systems, has been historically prescribed for managing various conditions, including ulcers, malignancies, hepatic diseases, and respiratory disorders [17]. Among its bioactive constituents, chelerythrine chloride (CH; Figure 1A), a benzophenanthridine alkaloid, has garnered significant scientific attention. Extensive investigations have revealed CH’s multifaceted pharmacological profile, encompassing antimicrobial, anticancer, and anti-inflammatory activities [18,19,20]. Fan et al. indicated that pretreatment with CH could decrease the lung tissue damage and inflammation in vivo [21]. Nevertheless, the underlying mechanism of it remains incompletely understood. This study aimed to evaluate the therapeutic effects of CH in lipopolysaccharide (LPS)-induced ALI in mice and its underlying mechanism. In the current investigation, we validated CH’s protective efficacy against LPS-induced ALI through direct administration in murine models. To unravel its mechanism of action, we engineered a photoaffinity probe CHP and implemented an integrated approach combining activity-based protein profiling (ABPP) for target identification and functional characterization.

## 2. Results

### 2.1. Effect of CH on Cytokine Production In Vitro

An excessive release of NO, TNF-α, IL-1β and IL-6 is closely associated with various inflammatory disorders [22]. To investigate the therapeutic potential of CH, we initially assessed its cellular toxicity in RAW264.7 macrophages through Cell Counting Kit-8 (CCK-8) assays. The results revealed that CH at 10 μM concentration did not induce significant cytotoxic effects (Figure 1B). Subsequently, we evaluated the modulatory effects of CH on inflammatory cytokine production in LPS-stimulated RAW264.7 macrophages at three concentrations (2.5, 5, and 10 μM) using ELISA. As shown in Figure 1C–F, 2.5 μM CH significantly inhibited the secretion of all measured cytokines, with the suppressive effects demonstrating a dose-dependent pattern.

### 2.2. Effect of CH on Nitric Oxide Synthase (iNOS) and Cyclooxygenase-2 (COX-2) Expression In Vitro

**Figure 2:**
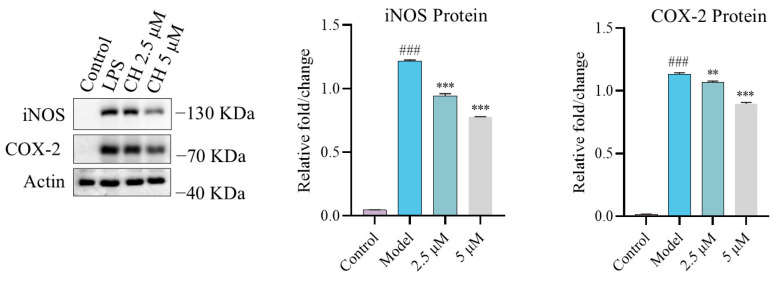
The effect of CH on iNOS and COX-2 expression. ### *p* < 0.001 vs. Control group; ** *p* < 0.01 vs. Model group; *** *p* < 0.001 vs. Model group.

Inducible iNOS emerges as the primary catalyst for NO production, whereas COX-2 governs the production of prostaglandins. iNOS and COX-2 overexpression correlates with chronic inflammation advancement [23]. To investigate CH’s modulatory effects in LPS-stimulated RAW264.7 macrophages, western blot (WB) analysis was carried out to assess its influence on iNOS and COX-2 expression profiles at concentrations of 2.5 and 5 μM. Our experimental data revealed that 2.5 μM CH effectively suppressed the LPS-induced overexpression of both proteins. Furthermore, this suppressive effect showed a clear correlation with increasing CH concentrations (Figure 2).

### 2.3. Therapeutic Impact of CH In Vivo

**Figure 3:**
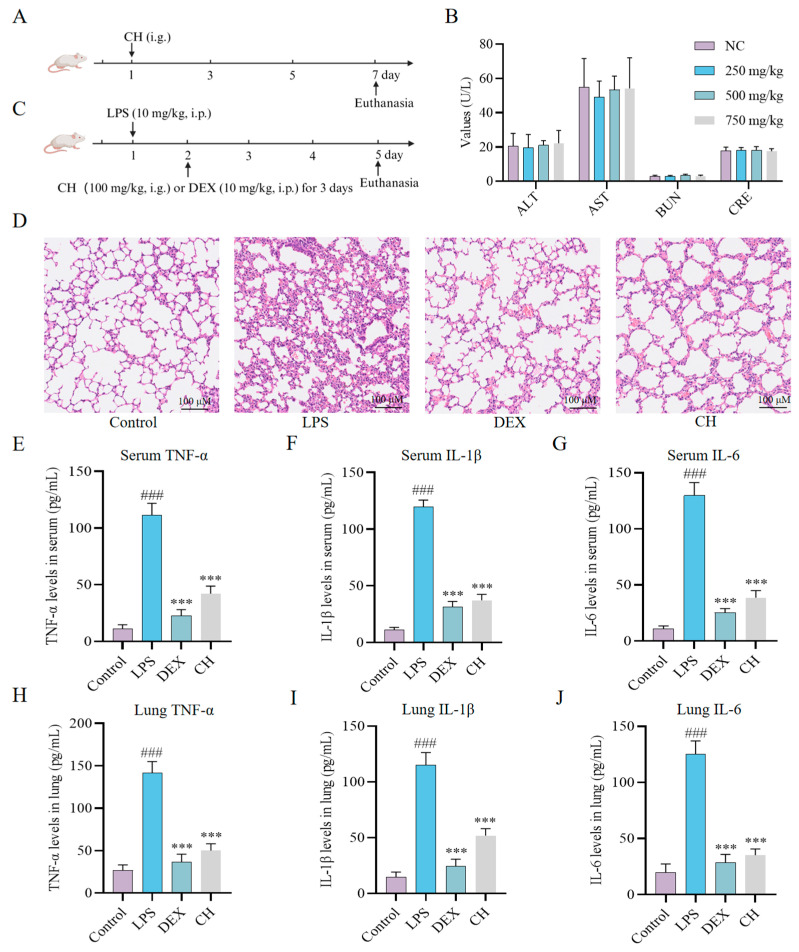
Alleviation effect of CH in vivo. Animal acute toxicity protocol (**A**) and test results (**B**) for CH. (**C**) Animal treatment protocol for CH. (**D**) Histological images of lung sections stained with H&E. Levels of inflammatory factors in serum (**E**–**G**) and lung tissue (**H**–**J**). ### *p* < 0.001 vs. Control group; *** *p* < 0.001 vs. LPS group. All experimental procedures were conducted with a minimum of three independent replicates.

To determine the safety profile of CH in vivo, acute toxicity test was employed (Figure 3A). CH exhibited excellent oral safety characteristics, with a half-lethal dose (LD_50_) exceeding 750 mg/kg. No significant abnormalities were observed in key functional indices, including blood urea nitrogen (BUN), alanine aminotransferase (ALT), aspartate aminotransferase (AST), and creatinine (CRE) (Figure 3B). Then, the therapeutic efficacy of CH was evaluated using an LPS-induced ALI mouse model, taking DEX as the positive control (Figure 3C). Hematoxylin-eosin (H&E) staining revealed the disruption of pulmonary architecture, pronounced thickening of alveolar septa, significant infiltration of inflammatory cells, alveolar hemorrhage, interstitial and intra-alveolar edema in the LPS group (Figure 3D). CH treatment notably mitigated the thickened alveolar septa and reduced edema (Figure 3D). Furthermore, while LPS administration markedly elevated serum and lung tissues concentrations of pro-inflammatory cytokines, CH intervention significantly attenuated these inflammatory responses (Figure 3E–J). These collective findings indicate that CH treatment effectively alleviates LPS-induced ALI in mice.

### 2.4. Synthesis and Bioactivity of the CH Probe

**Figure 4:**
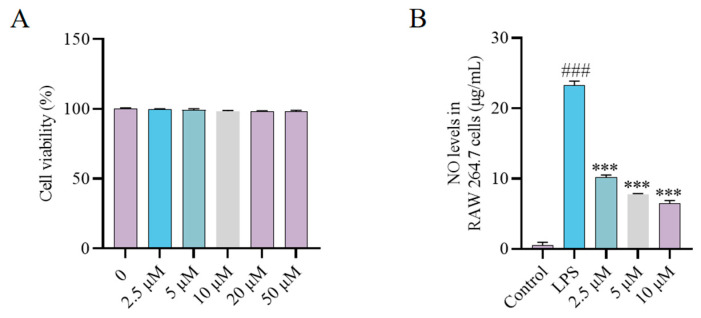
(**A**) Cytotoxic effect of CHP on RAW264.7 cells. (**B**) Inhibitory effect of CHP on NO release. ### *p* < 0.001 vs. Control group; *** *p* < 0.001 vs. LPS group. All experimental procedures were conducted with a minimum of three independent replicates.

ABPP has emerged as a powerful technique for direct drug targets identification, with the design of functional probes being a critical component of this approach. Therefore, a bioactive probe CHP was first synthesized for ABPP studies (Scheme S1). Cytotoxicity assessment revealed that CHP showed no toxic effects even at concentrations up to 50 μM (Figure 4A). Subsequently, the modulatory capacity of CHP (2.5, 5 and 10 μM) on LPS-triggered NO synthesis was evaluated in RAW264.7 macrophages using ELISA. The results revealed that 2.5 μM CH significantly suppressed NO secretion, with inhibition exhibiting a dose-dependent manner. Consequently, CHP was chosen for additional proteomic analysis, bioimaging research, and target identification studies.

### 2.5. Identifying the Binding Proteins of CH by ABPP

**Figure 5:**
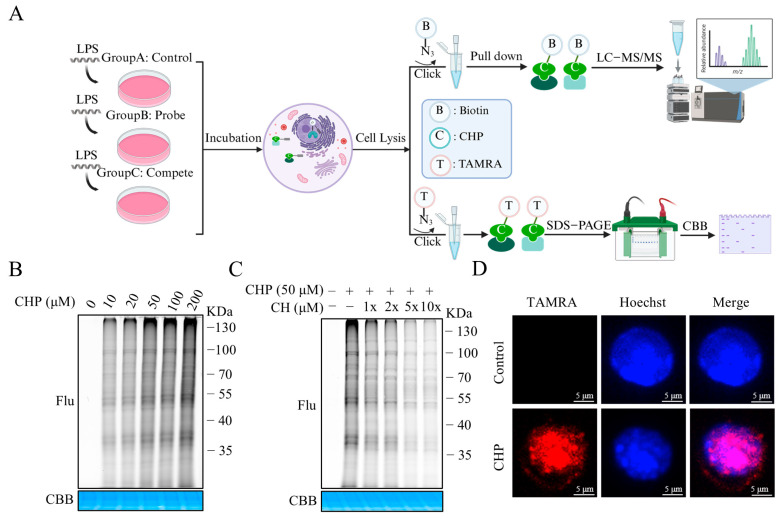
Identification target proteins of CH. (**A**) ABPP profiling workflow of CH. (**B**) Concentration-dependent CHP labelling in RAW264.7 cells. (**C**) Labeling of CHP proteins and competition between proteins labeling in RAW264.7 cells. (**D**) CHP co-localizes with RAW264.7 cells.

In situ targets fluorescence labeling in RAW264.7 cells of CHP at various concentrations was conducted (Figure 5A). The results demonstrated a concentration-dependent enhancement in fluorescence intensity, with optimal labeling efficiency achieved at 50 μM CHP (Figure 5B). Competitive binding studies showed that 50 μM CH pre-treatment effectively reduced CHP’s protein labeling capacity (Figure 5C). Immunofluorescence analysis further demonstrated that CHP could penetrate into cells, thereby enabling the labeling of target proteins within the cellular environment (Figure 5D). Following click chemistry conjugation with azide-biotin and subsequent affinity purification, we conducted dimethyl labeling-based quantitative proteomic analysis. Proteomic screening identified GAPDH, a crucial enzyme in the glycolytic pathway, as a high-confidence candidate among the upregulated proteins (Figure S1), prompting its selection for subsequent validation studies.

### 2.6. Validation of GAPDH as the Protein Target of CH

**Figure 6:**
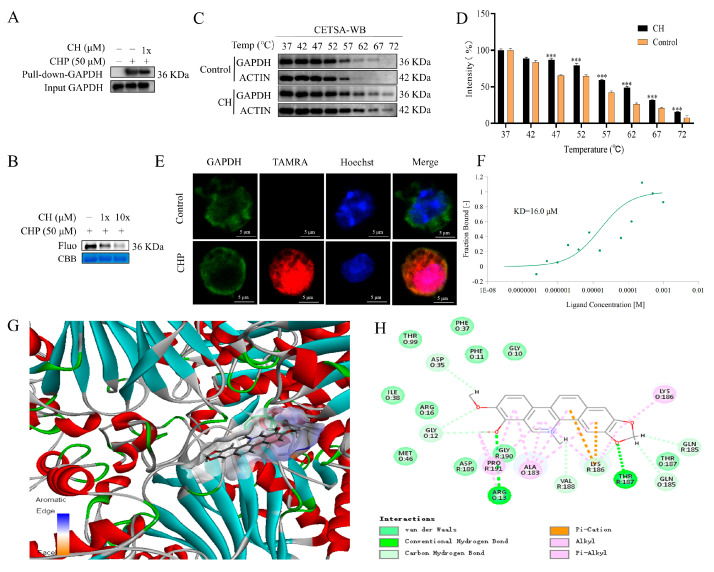
Validation of CH target proteins. (**A**) GAPDH was confirmed in experiments using a CHP pull-down assay combined with WB analysis. (**B**) Competition experiments with the pure protein GAPDH. (**C**,**D**) The interactions between CH and GAPDH were confirmed by CETSA-WB. *** *p* < 0.001 vs. Control group. (**E**) Immunofluorescence staining results of co-localization of GAPDH (green) and CHP (red). (**F**) The binding affinity of CH to GAPDH was quantified by MST. (**G**,**H**) Molecular docking of CH binding to GAPDH.

To validate GAPDH as the direct target of CH, we initially performed pull-down assays coupled with WB analysis in RAW264.7 cells. The results demonstrated specific binding between GAPDH and CHP, which could be competitively inhibited by CH pretreatment (Figure 6A). This competitive interaction was further confirmed in a purified GAPDH protein system, showing concentration-dependent displacement of CHP by CH (Figure 6B). Furthermore, the enhanced thermal stability of the GAPDH subsequent to CH binding was measured through cellular thermal shift assay (CETSA). Comparative analysis revealed enhanced thermal stability of CH-bound GAPDH protein relative to DMSO-treated controls (Figure 6C,D). In addition, the immunofluorescence experiment showed that CHP could co-localize with GAPDH and the nucleus in RAW264.7 cells (Figure 6E). For quantitative characterization of the CH and GAPDH interaction, we employed microscale thermophoresis (MST), which revealed strong binding affinity with a dissociation constant (KD) value of 16.0 μM (Figure 6F). Finally, Discovery Studio 4.5 was employed to conduct a molecular docking study between CH and GAPDH. As depicted in Figure 6G, CH could effectively fit within the active cavity of the binding site, achieving a libdock score of 102.71. The robust interactions are facilitated by hydrogen bonds with THR:187 and ARG:13, alongside van der Waals forces, pi–alkyl interactions, carbon–hydrogen interactions, and others (Figure 6H). Collectively, these multidisciplinary approaches provide compelling evidence for direct targeting of GAPDH by CH.

### 2.7. Inhibitory Effect of CH on the Glycolytic Pathway

**Figure 7:**
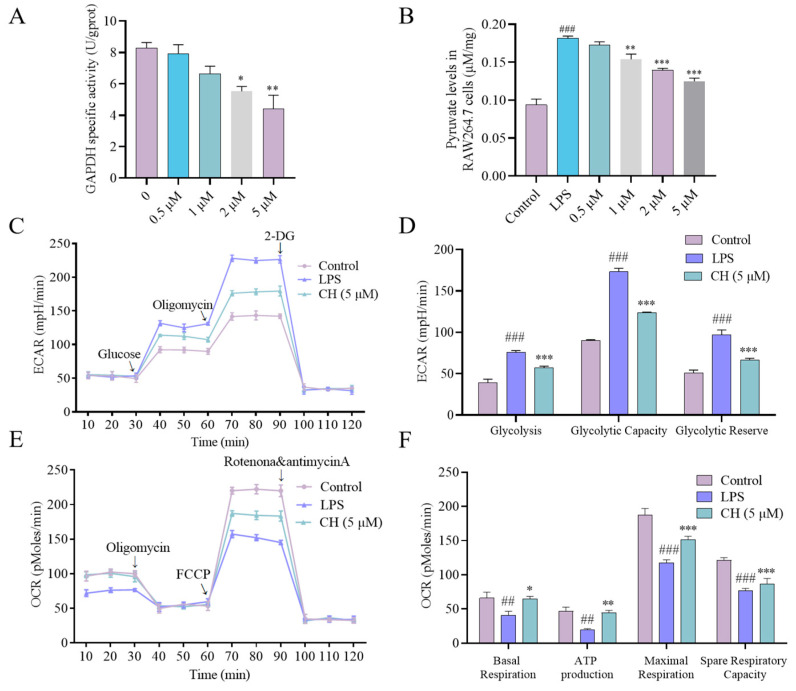
Inhibitory effect of CH on glycolytic pathway. (**A**) GAPDH enzyme activity was assayed after treating pure protein GAPDH for 3 h with CH. (**B**) CH reduced pyruvate levels in vitro. (**C**,**D**) ECAR and (**E**,**F**) OCR of RAW 264.7 cells untreated or treated with CH. ## *p* < 0.01, ### *p* < 0.001 vs. Control group; * *p* < 0.05, ** *p* < 0.01, *** *p* < 0.001 vs. LPS group. All experimental procedures were conducted with a minimum of three independent replicates.

**Figure 8:**
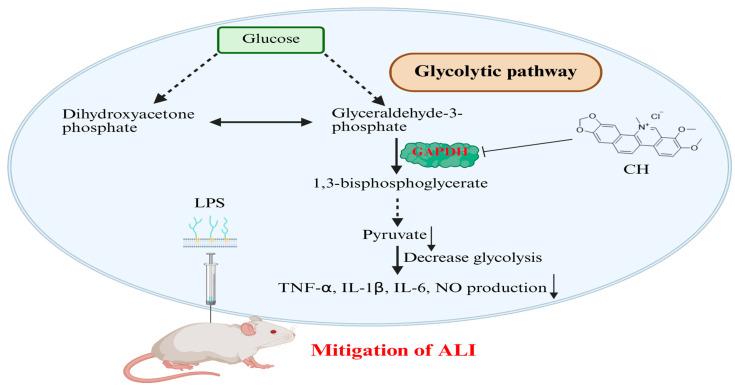
Schematic representation of CH ameliorating ALI in mice by inhibiting GAPDH-mediated glycolysis. LPS: lipopolysaccharide; TNF: tumor necrosis factor; IL: interleuki; NO: nitric oxide; GAPDH: glyceraldehyde-3-phosphate dehydrogenase.

As a key glycolytic regulator in activated macrophages [24], GAPDH’s enzymatic inhibition by CH was quantitatively assessed using a colorimetric assay kit. The results demonstrated dose-dependent suppression of GAPDH enzymatic activity at concentrations ranging from 0.5 to 5 μM (Figure 7A). Next, the cellular non-targeted metabolomics was employed to test the effect of CH-targeted GAPDH on pyruvate production in RAW264.7 cells. Results showed that pyruvate, a glycolysis-related metabolites downstream of GAPDH, was downregulated in a concentration-dependent manner (Figure 7B). In light of the demonstrated correlation between macrophage activation and metabolic reprogramming, where enhanced glycolysis correlates with increased extracellular acidification rate (ECAR) and decreased Oxygen Consumption Rate (OCR) [25,26], we examined CH’s impact on these metabolic parameters. As displayed in Figure 7C–F, the increased ECAR and decreased OCR induced by LPS were significantly alleviated by CH treatment in macrophage groups. The collective evidence suggest that CH exerts its anti-inflammatory effects by binding to and inhibiting GAPDH, which in turn reduces glycolysis, thereby alleviating LPS-induced ALI in mice (Figure 8).

## 3. Discussion

While a well-regulated inflammatory response is crucial for effective host defense against pathogens, disproportionate inflammation can lead to severe tissue damage and potentially fatal outcomes [2,3,4,27]. ALI is a pulmonary disorder marked by abnormal inflammation and pulmonary edema, which is caused by the damage to the alveolar–capillary barrier [28]. Uncontrolled inflammation is universally recognized as the principal pathogenic driver of ALI [29]. The inflammatory response is primarily mediated by cytokines, and the excessive accumulation of pro-inflammatory factors is associated with the pathophysiology of tissue damage in inflammatory diseases [30]. Currently, there are no clinically approved drugs that can effectively reduce the mortality rate of ALI [31]. The most effective treatment is drug intervention aimed at alleviating pulmonary and/or systemic inflammation, combined with supportive treatment based on mechanical ventilation [32].

Natural products with diverse anti-inflammatory properties and lung protective capacities, including flavonoids, alkaloids, and terpenoids, have emerged as promising therapeutic candidates for ALI treatment [33]. Among these, CH has attracted considerable attention for its anti-inflammatory potential. Emerging evidence suggests multiple mechanisms underlying CH’s protective effects: inhibition of NF-κB signaling in LPS-induced endotoxin shock [34], activation of the Nrf2/ARE pathway in bleomycin-induced pulmonary fibrosis [35], and modulation of the AMPK/mTOR/ULK-1 axis in rheumatoid arthritis fibroblast-like synoviocytes [16]. Previous work by Fan et al. demonstrated CH’s preventive efficacy against LPS-induced ALI through pre-treatment oral administration [21]. However, the therapeutic potential of direct CH administration in established ALI and its precise molecular targets remain unexplored.

As a pivotal glycolytic enzyme, GAPDH catalyzes the conversion of glyceraldehyde-3-phosphate to 1,3-bisphosphoglycerate, a rate-limiting step in glucose metabolism [36,37]. The glycolytic pathway is markedly upregulated in activated immune cells, with heightened GAPDH activity being a hallmark of macrophage activation [38,39,40]. Beyond its metabolic function, GAPDH also acts as an mRNA chaperone for various pro-inflammatory mediators in immune cells. Upon immune cell activation, GAPDH dissociates from these mRNA molecules, promoting their translation and leading to increased expression of inflammatory mediators [40,41]. Therefore, inhibiting the activity of GAPDH can effectively suppress glycolysis and subsequently alleviate the inflammatory response. Supporting this concept, previous studies have demonstrated that 4-Octyl itaconate exerts anti-inflammatory effects through GAPDH targeting [14], while dimethyl fumarate modulates immune responses via GAPDH-mediated glycolytic regulation [38].

Our current study provides novel insights into CH’s therapeutic profile. Treatment with 2.5 μM CH significantly suppressed the production of inflammatory mediators in LPS-activated macrophages. We present the first evidence of CH’s therapeutic efficacy in LPS-induced ALI in mice. However, current research revealed that CH at 20 μM still exhibits significant cytotoxicity, representing a major limitation for its clinical applications. Despite this, CH remains a promising lead compound for anti-ALI drug discovery. Rational structure–activity relationship studies could further optimize its pharmacological properties, potentially leading to more efficacious and safer derivatives.

We synthesized a biologically active chemical probe CHP and confirmed the binding of CH to GAPDH via ABPP. The direct interaction between CH and GAPDH was also validated through experiments, such as pull down-WB, CETSA, MST, and molecular docking. Furthermore, our findings demonstrated that CH dose-dependently inhibits GAPDH enzymatic activity at concentrations ranging from 0.5 to 5 μM, leading to reduced production of downstream pyruvate. Notably, CH exhibited no detectable cytotoxicity within this concentration range, and its inhibitory effect on GAPDH likely stems from direct enzyme binding. Previous studies have shown that enhanced glycolysis in macrophages increases ECAR while reducing OCR [25,26]. In LPS-stimulated RAW264.7 cells, CH treatment decreased ECAR and elevated OCR. However, this study still has some limitations. The study did not investigate whether CH alleviates LPS-induced ALI symptoms by inhibiting glycolysis through the targeting of GAPDH in vivo. The specific binding site of CH on GAPDH and its potential multi-target anti-inflammatory mechanisms remain to be fully elucidated.

In conclusion, we proved that CH inhibits the glycolysis pathway by targeting GAPDH, thereby alleviating LPS-induced ALI in mice. A comprehensive understanding of the treatment mechanism of CH for ALI has profound clinical significance, which may pave the way for discovering new treatment strategies and targets.

## 4. Materials and Methods

### 4.1. Agents and Chemicals

The supplier of DEX was Yuanye Bio-Technology Co., Ltd. (Shanghai, China). CH (98% pure) was commercially acquired from Herb Purify Co., Ltd. (Chengdu, China).

### 4.2. Cell Culture

The culture medium employed was DMEM (MeilunBio, Dalian, China), enriched with 10% FBS (MeilunBio, Dalian, China). Cell passage was performed at a split ratio of 1:3 when the confluence of RAW264.7 cells reached 80–90%. Afterward, the cells were maintained at 37 °C in a humidified 5% CO_2_ atmosphere.

### 4.3. Cell Viability Assay

Cell viability was determined using a CCK-8 (Beyotime, Shanghai, China) method. A suspension of RAW264.7 macrophages was dispensed into 96-well microplates (1 × 10^4^ cells per well). Following treatment of different concentrations of CH or CHP (2.5, 5, 10, 20 and 50 μM) for 24 h, the cells were cultured with 10 μL CCK-8 in each well at 37 °C for 2 h. Absorbance measurements were subsequently obtained using a multi-mode microplate reader at 450 nm (PerkinElmer, Waltham, MA, USA).

### 4.4. Detection of NO Production

Griess reaction kit (Beyotime, Shanghai, China) was used to determine the level of nitric oxide (NO) in the culture medium. Briefly, 1 × 10^4^ Raw 264.7 cells/well were seeded in a 96-well plate and maintained for 24 h. An amount of 2 μg/mL LPS was co-administered with or without CH/CHP at multiple concentrations (2.5, 5 and 10 μM) for 24 h at 37 °C. Then, 100 μL supernatant was collected from each well and followed by addition of 50 μL Griess Reagent I and 50 μL Griess Reagent II. Absorbance was measured at 540 nm.

### 4.5. Measurement of Inflammatory Factors

The levels of cytokines (TNF-α, IL-1β, and IL-6) in serum samples or cell supernatants were tested using ELISA kits (Jonlnbio, Shanghai, China). The samples were added to an enzyme-linked immunosorbent assay plate at 100 μL/well, with triplicate wells for each experimental group. The plate was sealed with an adhesive membrane and incubated at 37 °C for 1 h. Wells were aspirated and replenished with 100 μL biotin-conjugated antibody solution. The microplate was subsequently resealed and maintained at 37 °C for 1 h. Following antibody incubation, the wells were washed by adding 300 μL of wash buffer, incubating for 1 min, then removing the buffer and blotting the plate dry on absorbent paper. This washing procedure was repeated three times. Following this, a 100 μL aliquot of enzyme-conjugated working solution was distributed to all wells. After sealing, incubation proceeded at physiological temperature (37 °C) for 0.5 h. After discarding the solution, the washing steps were repeated five times. In the detection step, 90 μL of TMB substrate was added per well, then the plate was sealed and kept in darkness at 37 °C for 15 min. The enzymatic reaction was stopped by addition of 50 μL stop reagent to each well, with subsequent immediate optical density determination at 450 nm.

### 4.6. Western Blot

Cells were harvested and washed with 1 mL PBS to remove residual medium components. After triple PBS rinses, cellular proteins were extracted using 100 μL of Triton X-100-containing lysis buffer. Electrophoretic separation of proteins was performed using SDS-PAGE and subsequently transferred onto polyvinylidene fluoride membranes for immunoblot analysis. To prevent non-specific interactions, membranes were blocked with 5% BSA solution before overnight incubation at 4 °C with primary antibodies against COX-2 and iNOS (ABclonal, Wuhan, China). Following primary antibody incubation, membranes were exposed to species-specific secondary antibodies and incubated for 1 h. Enhanced chemiluminescence reagents were used to visualize protein bands, which were then detected by an imaging system (Azure C400, Azure Biosystems, Inc., Dublin, CA, USA).

### 4.7. Acute Toxicity in Mice

Ethical approval for all animal procedures was granted by China Animal Care in conjunction with the China Academy of Chinese Medical Sciences. Kunming mice (20.0 ± 1.0 g, 6-weeks old) were utilized. In a single-dosing trial, 250, 500, or 750 mg/kg of CH were administered orally. For seven days, the body weight and survival were continuously tracked. Zhongsheng Beikong Biotechnology (Beijing, China) detection kits were used to examine the ALT, AST, BUN, and CRE levels using a Hitachi 7170 chemistry analyzer (Hitachi, Tokyo, Japan).

### 4.8. LPS-Induced ALI Mouse Model

Ethical approval for all animal procedures was granted by China Animal Care in conjunction with the China Academy of Chinese Medical Sciences. Forty male BALB/c mice (8-weeks old) were obtained from the Department of Medicine, Peking University. These mice were provided with a standard diet and water for a one-week period to allow for acclimatization. After the initial period, randomization was performed to create four experimental groups (*n* = 10/group): Control (saline treatment), LPS-induced ALI model, DEX treatment (10 mg/kg/day, intraperitoneal injection) and CH treatment (100 mg/kg/day, oral gavage). Except for the control group, the remaining groups were administered for three consecutive days after 24 h of LPS induction. Blood samples were collected from the eyes, and lung tissues were preserved in 4% paraformaldehyde solution for subsequent H&E staining.

### 4.9. Fluorescent Labeling and Competition

RAW264.7 cells were spread at a density of 5 × 10^5^ per well in a 6-well plate, with 2 mL of cell suspension per well. After adding LPS at a final concentration of 2 μg/mL, the cells were induced for 10 h. Following a 2 h treatment with CH at 37 °C (or no treatment), cells were incubated with CHP for 2 h. Following incubation with CHP at 37 °C for 2 h, cells underwent 2 h of CH treatment or no treatment. For optimal cell lysis, cells were treated with 100 μL Triton-based lysis buffer supplemented with protease inhibitors and subjected to ultrasonic disruption, followed by 20 min exposure to 365 nm ultraviolet radiation. They were then centrifuged at 4 °C for 10 min to separate soluble protein components and standardize protein concentration and incubated in a click chemistry reaction mixture with final concentrations of CuSO_4_ (1 mM), Tcep (1 mM), THPTA (100 μM) and TAMRA-N_3_ (50 μM) at 37 °C for 1 h. Protein precipitation was achieved by adding 1 mL of pre-cooled acetone, followed by the removal of the supernatant and evaporation of the residue. The protein pellet was resuspended in loading buffer and sonicated to ensure solubilization before subjecting it to 10% SDS-PAGE gel electrophoresis after a heating step at 95 °C for 10 min. The highly pure GAPDH protein (MedChemExpress, Monmouth Junction, NJ, USA) was diluted to a final concentration of 1 μg/μL, with 6 μg per group, and subsequently handled in a manner consistent with the previous steps. For visualization, fluorescence scanning was carried out using a laser scanner, while coomassie brilliant blue staining served as a reference for protein-loading control.

### 4.10. Target Identification and Pull Down

The cultured RAW264.7 cells were treated with LPS (2 μg/mL) for 10 h, followed by incubation with 50 μM CH for 2 h at 37 °C. Then, 50 μM CHP was added, and the cells were further incubated for 2 h at 37 °C for subsequent treatment. The lysed cells were harvested and prepared for the click reaction, ensuring consistent protein quantification throughout the process. After acetone precipitation to isolate the proteins, the precipitates were washed with 1 mL of pre-cooled methanol and resuspended in 1 mL of 1.2% SDS/PBS solution. Protein dispersion was achieved through sonication, followed by a 10 min boiling step at 95 °C. The samples were then centrifuged at 4 °C for 10 min, after which the supernatant was collected and diluted in 6 mL 0.2% SDS/PBS. The samples were incubated with 70 μL of streptavidin affinity magnetic beads and washed with sequential washes with 5 mL of 1% SDS/PBS, 0.1% SDS/PBS, and 6 M urea, respectively. Alkylation was performed by adding DTT and IAA to a target concentration of 10 mM, followed by overnight digestion with trypsin at a 1:100 ratio at 37 °C. A 200 μL volume of 25 mM ammonium bicarbonate solution was added, followed by introduction of formic acid to reach a 0.1% final concentration. The samples were then labeled using 24 μL of 0.6 M dimethylation prior to analysis by LC-MS/MS [42].

### 4.11. Cellular Thermal Shift Assay

CH or DMSO was added to 1 mL of RAW264.7 cell lysate (1 mg/mL) followed by 2 h incubation at 37 °C. Then, an equal volume (50 μL) of treated lysate was aliquoted into individual PCR tubes. Using a PCR analyzer (Thermo Fisher Scientific, Waltham, MA, USA), the tubes underwent 3 min heat treatment with thermal cycling across a 37 °C to 72 °C gradient. The tubes were centrifuged for 10 min at 4 °C. The resulting protein lysate was further analyzed by western blot to assess protein expression [43].

### 4.12. Fluorescence Co-Localization

To investigate the co-localization of CHP with RAW264.7 cells and GAPDH protein, the cells were seeded at a density of 2 × 10^4^ in four compartment glass-bottomed petri dishes (500 μL suspension per well). CHP-treated cells (50 μM, 37 °C, 2 h) were subsequently irradiated using 365 nm UV light (20 min exposure). Membrane permeabilization was achieved with 0.2% Triton X-100, and the CHP-labeled GAPDH samples were blocked with 5% BSA for 1 h. The click chemistry reaction components were subsequently introduced to the cellular system following the previously described protocol. According to the experimental grouping, incubate overnight at 4 °C with or without GAPDH specific primary antibody. The cells were incubated with a secondary antibody for 1 h. To visualize the cell nuclei, hoechst staining (final concentration 0.5 μg/mL) was performed for 10 min. Finally, the dishes were imaged using a fluorescence microscope (Leica TCS SP8 SR, Wetzlar, Germany) to capture the co-localization pattern of CHP with RAW264.7 cells and intracellular GAPDH [44].

### 4.13. Microscale Thermophoresis

The purified GAPDH protein was acquired from MedChemExpress and diluted to a concentration of 1 μg/μL. Protein samples (25 μg) were combined with 350 μL PBST and 4 μL of kit supplied dye (NanoTemper, Munich, Germany), followed by 0.5 h ice incubation. CH was diluted at a halved concentration starting from 2 mM to the predetermined concentration gradient. The diluted CH and the pure GAPDH protein were combined at a 1:1 ratio. Subsequently, the prepared samples were drawn into the detection instrument using a capillary tube for analysis. The method was applied to determine the binding affinity of RAW264.7 cells towards CH [45].

### 4.14. Assessment of GAPDH Enzymatic Activity

Different concentrations of CH were incubated with 10 μg of GAPDH at 37 °C for 3 h. An amount of 40 μL aliquots of both standard solutions and experimental samples were aliquoted into ELISA plate wells, strictly adhering to the manufacturer’s protocol (Colorimetric Assay Kit, Elabscience, Wuhan, China). Each well received 100 μL of working solution supplemented with 20 μL chromogenic substrate. After brief vortex mixing (5 s), initial absorbance at 450 nm was measured by ELISA microplate reader. Following 10 min dark incubation at 37 °C, the plate was reanalyzed at 450 nm to quantify OD value changes.

### 4.15. Measurement of Pyruvate Concentration

The 6-well plates were inoculated with RAW264.7 macrophages (5 × 10^5^ cells per well) and subsequently exposed to LPS (1 μg/mL) combined with varying doses of CH for 3 h at 37 °C. After incubation, the samples were collected, and pyruvate levels were quantified using a kit from Shanghai Enzyme Link Biological Co., Ltd of Shanghai, China. For protein extraction, 80 μL lysis buffer was added and subjected to sonication. After 0.5 h standing, samples were centrifuged (8000× *g*, 10 min, RT) and supernatants collected for analysis. Subsequently, 75 μL aliquots of both test samples and standards were combined with 25 μL kit-provided reagent 1, agitated briefly using a vortex mixer prior to a 2 min RT incubation. Following addition of 125 μL reagent 2 and thorough mixing, absorbance was measured at 520 nm.

### 4.16. Seahorse Extracellular Flux Analyser Assays for OCR and ECAR

XF24-well culture plates were seeded with 10^4^ RAW264.7 cells per well and adhered for 24 h. Cells were rinsed twice with XF base media devoid of sodium bicarbonate, brought to a pH of 7.4, 10 mM glucose added, and then incubated for one hour at 37 °C in a non-CO_2_ incubator. In order to measure OCR and ECAR using the Seahorse XF24 Analyzer (Agilent Technologies, Santa Clara, CA, USA), chemicals that alter oligomycin (1 μM), FCCP (1 μM), antimycin A (1 μM) and rotenone (0.5 μM) were consecutively injected as part of a mitochondrial stress test. Wave software 2.6.3 (Agilent Technologies, Santa Clara, USA) was used to evaluate the data [46].

### 4.17. Molecular Docking

The GAPDH’s crystal structure (code 6M61) was downloaded from the Protein Data Bank [47]. Discovery Studio 4.5 was employed to construct the ligand structures and to conduct energy minimization procedures. Prior to the docking process, all ligands and water molecules were removed from the protein structure, which was then refined using alternate conformations. The top-scoring docking conformation was selected for further analysis of receptor–ligand interactions using the Discovery Studio 4.5 software [48].

### 4.18. Quantification and Statistical Analysis

The means ± SEM of at least three separate experiments were used to express the data. The evaluation of the data was conducted with Graphpad Prism 9.5. The significance of differences was evaluated using either a one-way ANOVA followed by Tukey’s test in multiple groups or a Student’s *t*-test between groups. Statistical significance is defined as a *p*-value of less than 0.05.

## Data Availability

The datasets generated during the current study are available from the corresponding author on reasonable request. Clinical trial number: not applicable.

## Supplementary Materials

The following supporting information can be downloaded at: https://www.mdpi.com/article/10.3390/molecules30122572/s1, Scheme S1: The synthetic route of CHP; Figure S1: Quantitative mass spectrometry-based profiling of CHP (50 μM) in the presence of CH (50 μM).

## Abbreviations

The following abbreviations are used in this manuscript:

ABPP	activity-based protein profiling
ALI	acute lung injury
ALT	alanine aminotransferase
AST	aspartate aminotransferase
BUN	blood urea nitrogen
CETSA	cellular thermal shift assay
CH	chelerythrine chloride
COX-2	cyclooxygenase-2
CRE	creatinine
DEX	dexamethasone
ECAR	extracellular acidification rate
GAPDH	glyceraldehyde-3-phosphate dehydrogenase
H&E	hematoxylin-eosin staining
IL	interleukin
iNOS	nitric oxide synthase
KD	dissociation constant
LD_50_	half-lethal dose
LPS	lipopolysaccharide
MST	microscale thermophoresis
NO	nitric oxide
OCR	oxygen consumption rate
TNF	tumor necrosis factor
WB	western blot
