# Supplementary material for: Chelerythrine Chloride Alleviated Lipopolysaccharide-Induced Acute Lung Injury by Inhibiting Glycolytic Pathway Through Targeting Glyceraldehyde-3-Phosphate Dehydrogenase
Source: Molecules. 2025 Jun 12;30(12):2572. doi: 10.3390/molecules30122572 (PMC12195816; doi:10.3390/molecules30122572)
Supplement: Supplementary file 1 [file molecules-30-02572-s001.zip › molecules-3625287-supplementary.pdf]

Article

Chelerythrine Chloride Alleviated Lipopolysaccharide-Induced Acute Lung Injury by Inhibiting Glycolytic Pathway Through Targeting Glyceraldehyde-3-Phosphate Dehydrogenase

Yuting He [1,2,†], Tianyun Fan [3,†], Ruishen Zhuge [3], Huiying Li [3], Guanjun Li [3], Lirun Zhou [3], Liting Xu [3], Xiaojiang Hao [1,2], Wei Gu [1,2,*] and Jigang Wang [3,*]

[1] State Key Laboratory of Discovery and Utilization of Functional Components in Traditional Chinese Medicine, Guizhou Medical University, Guiyang 550014, China; 18323186786@163.com (Y.H.); haoxj@mail.kib.ac.cn (X.H.)
[2] Natural Products Research Center of Guizhou Province, Guiyang 550014, China
[3] State Key Laboratory for Quality Ensurance and Sustainable Use of Dao-di Herbs, Artemisinin Research Center, and Institute of Chinese Materia Medica, China Academy of Chinese Medical Sciences, Beijing 100700, China; fty1668@163.com (T.F.); zhugedentist@pku.edu.cn (R.Z.); lihuiying1993@126.com (H.L.); liguanjun0201@sina.com (G.L.); lirunzhou1997@163.com (L.Z.); 15910456090@163.com (L.X.)
* Correspondence: guwei2009@126.com (W.G.); jgwang@icmm.ac.cn (J.W.)
† These authors made equal contribution to this work.

Academic Editor: Míchel Frédérich

Received: 19 April 2025
Revised: 5 June 2025
Accepted: 10 June 2025
Published: 12 June 2025

Citation: He, Y.; Fan, T.; Zhuge, R.; Li, H.; Li, G.; Zhou, L.; Xu, L.; Hao, X.; Gu, W.; Wang, J. Chelerythrine Chloride Alleviated Lipopolysaccharide-Induced Acute Lung Injury by Inhibiting Glycolytic Pathway Through Targeting Glyceraldehyde-3-Phosphate Dehydrogenase. *Molecules* **2025**, *30*, 2572. https://doi.org/10.3390/molecules30122572

Copyright: © 2025 by the authors. Licensee MDPI, Basel, Switzerland. This article is an open access article distributed under the terms and conditions of the Creative Commons Attribution (CC BY) license (https://creativecommons.org/licenses/by/4.0/).

The synthetic route of CHP was presented in Scheme S1.

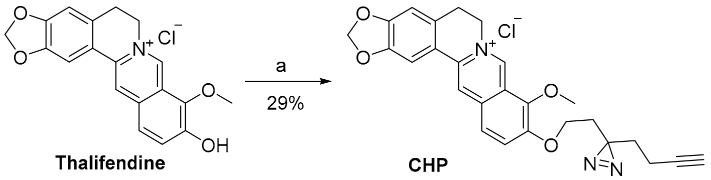

Scheme S1. (a) 3-(but-3-yn-1-yl)-3-(2-iodoethyl)-3H-diazirine, K_2CO_3, CH_3CN, 71 °C, 5 h.

Thalifendine (2.0 mmol), 3-(but-3-yn-1-yl)-3-(2-iodoethyl)-3H-diazirine (3.2 mmol) and K_2CO_3 (3.2 mmol) were reacted at 71 ℃ for 5 h in anhydrous CH_3CN.. Then, cooled and filtered the reaction mixture, and the residue was purified by CH_2Cl_2 and CH_3OH to obtain CHP in 29% yield. A yellow solid; m.p.: 191-193 °C; ^{1}H NMR: δ 9.90 (s, 1H), 8.93 (s, 1H), 8.16 (d, *J* = 9.0 Hz, 1H), 7.97 (d, *J* = 9.0 Hz, 1H), 7.80 (s, 1H), 7.09 (s, 1H), 6.18 (s, 2H), 4.93 (t, *J* = 6.6 Hz, 2H), 4.19 (s, 3H), 4.16 (t, *J* = 6.0 Hz, 2H), 3.21 (t, *J* = 6.6 Hz, 2H), 2.85 (t, *J* = 2.4 Hz, 1H), 2.08 (td, *J* = 7.2, 2.4 Hz, 2H), 2.02 (t, *J* = 6.0 Hz, 2H), 1.74 (t, *J* = 7.2 Hz, 2H); ^{13}C NMR: δ 149.9, 149.2, 147.7, 145.5, 143.8, 137.6, 133.2, 130.7, 127.4, 123.4, 121.5, 120.4, 120.2, 108.4, 105.4, 102.1, 83.2, 71.8, 64.6, 62.1, 55.2, 32.1, 31.5, 26.9, 26.3, 12.6; HRMS: calcd for $C_{26}H_{24}N_3O_4Cl$ [M-Cl]$^+$: 442.1761, found: 442.1758.

CHP: ^{1}H NMR, ^{13}C NMR, HRMS and HPLC spectra.

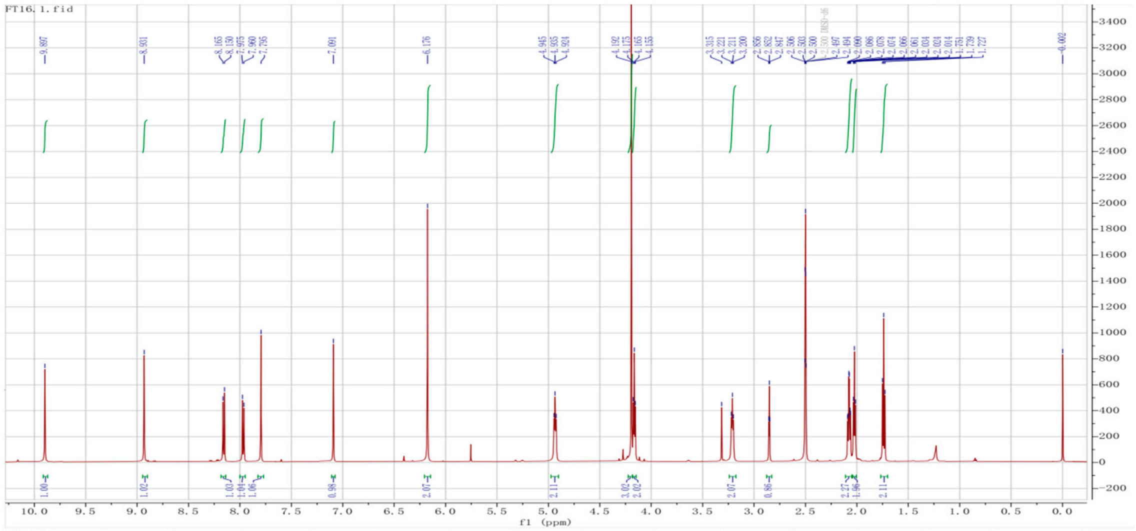
FT16.1.fid
9.897
8.931
7.795
7.091
6.176
-0.002
f1 (ppm)

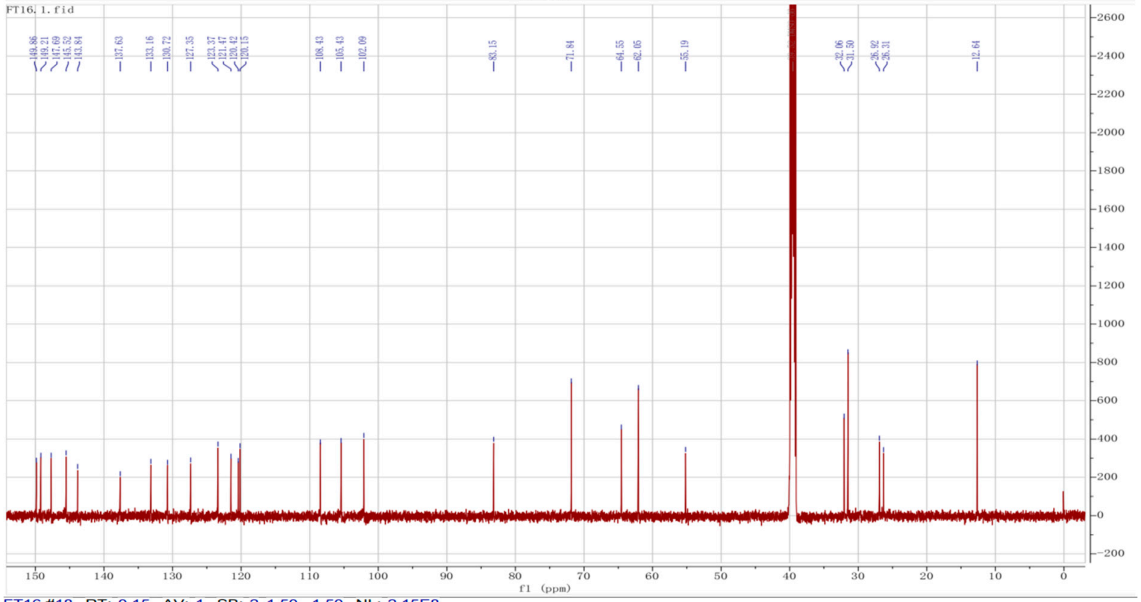
FT16.1.fid
149.86
149.21
147.69
145.52
143.84
137.63
133.16
130.72
127.35
123.37
121.47
120.42
120.15
108.43
105.43
102.09
83.15
71.84
64.55
62.05
55.19
32.06
31.50
26.92
26.31
12.64
f1 (ppm)

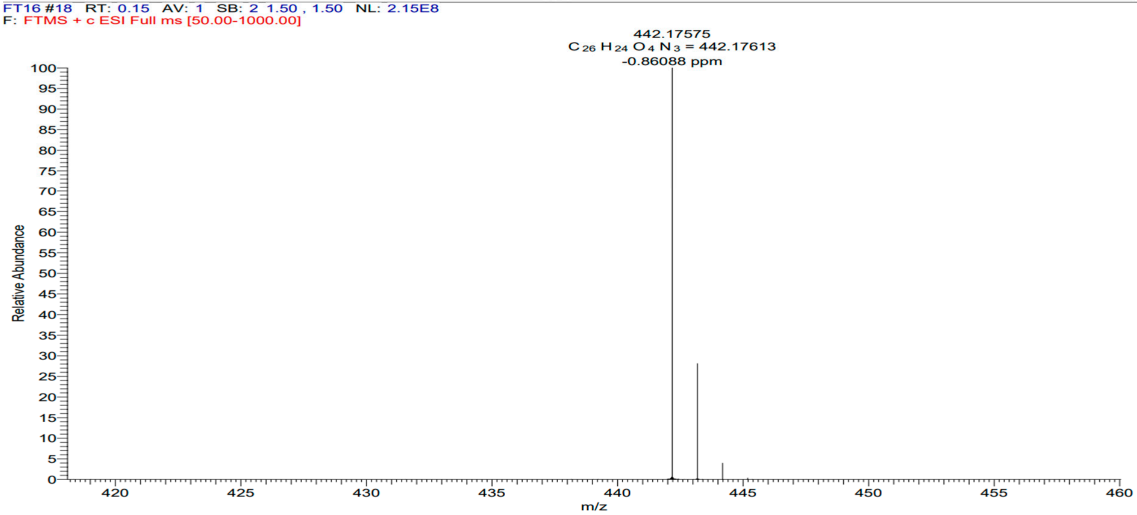
FT16 #18 RT: 0.15 AV: 1 SB: 2 1.50 , 1.50 NL: 2.15E8
F: FTMS + c ESI Full ms [50.00-1000.00]
442.17575
C 26 H 24 O 4 N 3 = 442.17613
-0.86088 ppm
Relative Abundance
m/z

A

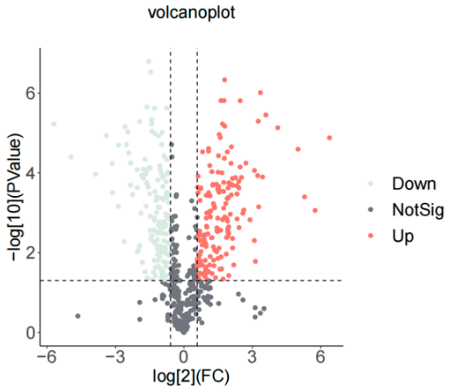

Figure S1. Quantitative mass spectrometry-based profiling of CHP (50 μM) in the presence of CH (50 μM).
